# Biomonitoring of Mycotoxins in Plasma of Patients with Alzheimer’s and Parkinson’s Disease

**DOI:** 10.3390/toxins13070477

**Published:** 2021-07-10

**Authors:** Beatriz Arce-López, Lydia Alvarez-Erviti, Barbara De Santis, María Izco, Silvia López-Calvo, Maria Eugenia Marzo-Sola, Francesca Debegnach, Elena Lizarraga, Adela López de Cerain, Elena González-Peñas, Ariane Vettorazzi

**Affiliations:** 1 Department of Pharmaceutical Technology and Chemistry, Research Group MITOX, School of Pharmacy and Nutrition, Universidad de Navarra, 31008 Pamplona, Spain; barce@alumni.unav.es (B.A.-L.); elizarraga@unav.es (E.L.); mgpenas@unav.es (E.G.-P.); 2 Laboratory of Molecular Neurobiology, Center for Biomedical Research of La Rioja (CIBIR), Piqueras 98, 3rd Floor, 26006 Logroño, Spain; laerviti@riojasalud.es (L.A.-E.); mizco@riojasalud.es (M.I.); 3 National Reference Laboratory for Mycotoxins and Plant Toxins, Istituto Superiore di Sanità, 00161 Roma, Italy; barbara.desantis@iss.it (B.D.S.); francesca.debegnach@iss.it (F.D.); 4 Servicio de Neurología, Hospital San Pedro, Piqueras 98, 26006 Logroño, Spain; slcalvo@riojasalud.es (S.L.-C.); memarzo@riojasalud.es (M.E.M.-S.); 5 Department of Pharmacology and Toxicology, Research Group MITOX, School of Pharmacy and Nutrition, Universidad de Navarra, 31008 Pamplona, Spain; acerain@unav.es; 6 IdiSNA, Navarra Institute for Health Research, 31008 Pamplona, Spain

**Keywords:** mycotoxins, ochratoxin A, sterigmatocystin, human exposure, Parkinson´s disease, Alzheimer´s disease, neurodegenerative disease

## Abstract

Exposure to environmental contaminants might play an important role in neurodegenerative disease pathogenesis, such as Parkinson´s disease (PD) and Alzheimer´s disease (AD). For the first time in Spain, the plasmatic levels of 19 mycotoxins from patients diagnosed with a neurodegenerative disease (44 PD and 24 AD) and from their healthy companions (25) from La Rioja region were analyzed. The studied mycotoxins were aflatoxins B1, B2, G1, G2 and M1, T-2 and HT-2, ochratoxins A (OTA) and B (OTB), zearalenone, sterigmatocystin (STER), nivalenol, deoxynivalenol, 3-acetyldeoxynivalenol, 15-acetyldeoxynivalenol, deepoxy-deoxynivalenol, neosolaniol, diacetoxyscirpenol and fusarenon-X. Samples were analyzed by LC-MS/MS before and after treatment with β-glucuronidase/arylsulfatase in order to detect potential metabolites. Only OTA, OTB and STER were detected in the samples. OTA was present before (77% of the samples) and after (89%) the enzymatic treatment, while OTB was only detectable before (13%). Statistically significant differences in OTA between healthy companions and patients were observed but the observed differences might seem more related to gender (OTA levels higher in men, *p*-value = 0.0014) than the disease itself. STER appeared only after enzymatic treatment (88%). Statistical analysis on STER, showed distributions always different between healthy controls and patients (patients’ group > controls, *p*-value < 0.0001). Surprisingly, STER levels weakly correlated positively with age in women (rho = 0.3384), while OTA correlation showed a decrease of levels with age especially in the men with PD (rho = −0.4643).

## 1. Introduction

Neurodegenerative diseases are one of the most frequent pathologies associated with aging, being Alzheimer’s disease (AD) the most common, and Parkinson’s disease (PD) the second most common. AD is the most prevalent form of dementia causing a progressive impairment in memory and other cognitive functions. AD is characterized pathologically by the presence of neurofibrillary tangles which contain hyperphosphorylated Tau, extracellular aggregates of amyloid β and severe neuronal loss in the brain [1]. PD is characterized clinically by motor symptoms such as bradykinesia, rigidity, resting tremor and postural instability, and non-motor symptoms such as olfactory dysfunction, constipation, depression, sleep disorders, pain and fatigue. PD is characterized pathologically by the loss of dopaminergic neurons in the substantia nigra *pars compacta* and the presence of Lewy bodies which contain aggregates of alpha-synuclein protein [2]. 

Despite extensive research, the mechanism underlying the cause in the majority of AD and PD cases remains unknown. Dominantly-inherited Alzheimer’s disease is relatively rare and in more than 90% of patients AD etiology is driven by a combination of genetic and environmental factors [3]. The majority of PD cases are sporadic and the familial of monogenic PD forms represent about 10% of PD cases [4]. Although the precise mechanism of neurodegeneration in AD and PD is not clear, there is likely a complex etiology involving multiple environmental, age-related, genetic, epigenetic and inflammatory factors [5,6].

It is widely accepted that the etiology of AD is multifactorial and its pathogenesis is influenced by the interaction of numerous factors, including environmental factors, lifestyle and genetics elements [7,8]. The association between environmental factors and AD has attracted considerable attention recently. Certain environmental factors, such as metals [9], air pollution [10], pesticides [11] or biotoxins produced by bacteria, molds and viruses [12], have been reported to increase the risk of AD and play a crucial role in the onset and progression of AD through several pathological mechanisms [7,8,13]. 

In the case of PD, the fact that the disease is accompanied by other non-motor symptoms in organs highly exposed to the environment (olfactory and gastrointestinal systems) reinforces the hypotheses of the role of environmental factors in the etiology of PD. Many chemicals such as pesticides, metals (iron and lead), polychlorinated biphenyls, solvents such as trichloroethylene, perchloroethylene as well a traffic particles have been linked to PD [14,15]. Among them, the stronger evidence points to pesticides [16]. Indeed, it is known that people living in rural areas, exposed to neurotoxins present in crops, and well and spring waters have been shown to be at higher risk of developing PD [17,18]. 

Another group of compounds present in rural areas long before pesticides are mycotoxins. Mycotoxins are naturally-occurring contaminants produced by different fungal species such as *Aspergillus, Penicillium and Fusarium*, that contaminate crops, mainly cereals, nuts and vegetables worldwide. The economical agricultural losses are huge [19]; however, the main concern is related with human and farm animal health. They are known to cause severe and long-term diseases related with their hepatotoxic, nephrotoxic, immunotoxic, genotoxic and carcinogenic properties as well as their deleterious effects on the endocrine or reproductive systems [20,21,22]. Indeed, due to their toxic effects maximum limits in different foodstuffs, foods and feed have been laid down at EU level [23,24,25].

Nowadays there is an increasing awareness for minimizing mycotoxins exposure [22] due to the fact that: (i) the general population is widely exposed to mycotoxins mainly through diet, with a worldwide occurrence in foodstuffs above the detectable levels, being up to 60–80% [26], (ii) human and animal are exposed to more than one mycotoxin (cocktails of mycotoxins) and (iii) climate change might increase the risk for mycotoxin contamination in some areas. 

This awareness has also led to an increased need to carry out analysis of mycotoxins in biological fluids through human biomonitoring (HBM) in order to know the real exposure of animals and humans. Indeed, HBM is promoted as an essential complement to direct mycotoxin determination in food [27,28].

Although, as mentioned, the scientific literature has many publications describing the several deleterious effects of the different mycotoxins or quantifying the levels of mycotoxins in foods, little has been done regarding its quantification in human samples and they have been practically unexplored as etiological agents of neurodegenerative diseases. 

Very few studies have focused on this purpose. Some case studies from patients diagnosed with AD and related with a chronic inflammatory response syndrome (CIRS), have directly pointed to mycotoxin exposure via inhalation in moldy environments as the cause of the disease [29]. Some authors have also hypothesized about a link between diet and neurodegeneration with the involvement of bacterial lipopolysaccarides and fungal mycotoxins in amyloid beta (Aβ) homeostasis, a process related to AD [30].

More recently, in vitro and in vivo studies carried out by our group [31] demonstrated that subchronic exposure to the mycotoxin ochratoxin A (OTA) induced some of the key pathological features of PD such as loss of striatal dopaminergic innervation and dopaminergic cell dysfunction accompanied with motor impairments and increased phosphorylated alpha-synuclein levels. 

All this information as well as the need for continuous HBM, led us to quantify the levels of mycotoxins in healthy donors and patients with neurodegenerative diseases such as PD or AD, from a region of Northern Spain: La Rioja. 

Therefore, in the present study the levels of 19 mycotoxins of major risk for human health and some of their metabolites have been quantitatively analyzed by high-performance liquid chromatography-mass spectrometry (LC-MS/MS): aflatoxins B1 (AFB1), B2 (AFB2), G1 (AFG1), G2 (AFG2), M1 (AFM1), ochratoxin A (OTA) and B (OTB), sterigmatocystin (STER), deoxynivalenol (DON), 3-acetyldeoxynivalenol (3-ADON), 15-acetyldeoxynivalenol (15-ADON), deepoxy-deoxynivalenol (DOM-1), diacetoxyscirpenol (DAS), nivalenol (NIV), fusarenon-X (FUS-X), neosolaniol (NEO), zearalenone (ZEA), T-2 and HT-2.

Moreover, due to the fact that mycotoxins can be metabolized, plasma samples have been analyzed before and after treatment with a mixture of β-glucuronidase and arylsulfatase in order to study, in an indirect way, the presence of glucuronide or sulfate conjugate metabolites of the studied mycotoxins. This is the first time that this analysis has been conducted on plasma from patients diagnosed with AD or PD. Data from healthy donors, companions of the patients, are also included. The age of the patients and the disease stage, diagnosed by experienced neurologists and based on Hoehn and Yahr (HY) scale for PD and Global Deterioration Scale (GDS) for AD, have been matched with the samples.

## 2. Results and Discussion

### 2.1. LC-MS/MS Analysis

Due to their different physicochemical characteristics, mycotoxins were divided for chromatographic separation into two groups (see material and methods section). 

**Figure 1:**
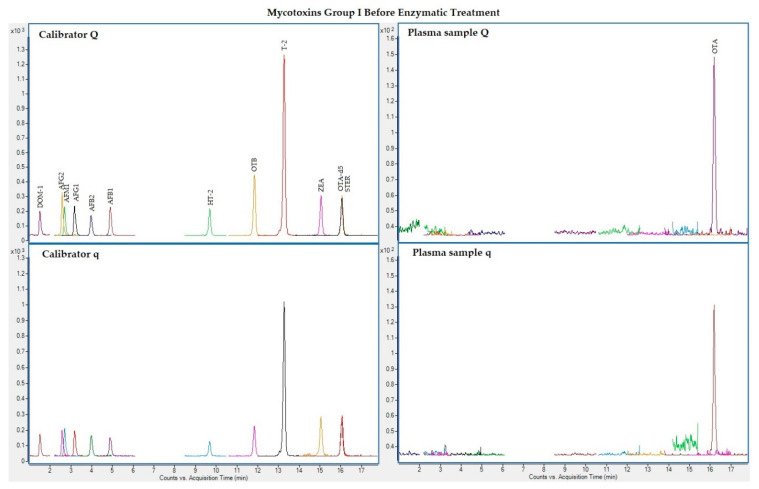
Superposed extracted chromatograms for mycotoxins group I (DOM-1, AFG2, AFM1, AFG1, AFB2, AFB1, OTB, ZEA, STER, OTA, T-2, HT-2) before enzymatic treatment. Calibrator: 10xLOQ level. Plasma sample: code 1-4. Q (quantification) and q (qualification) transitions.

**Figure 2:**
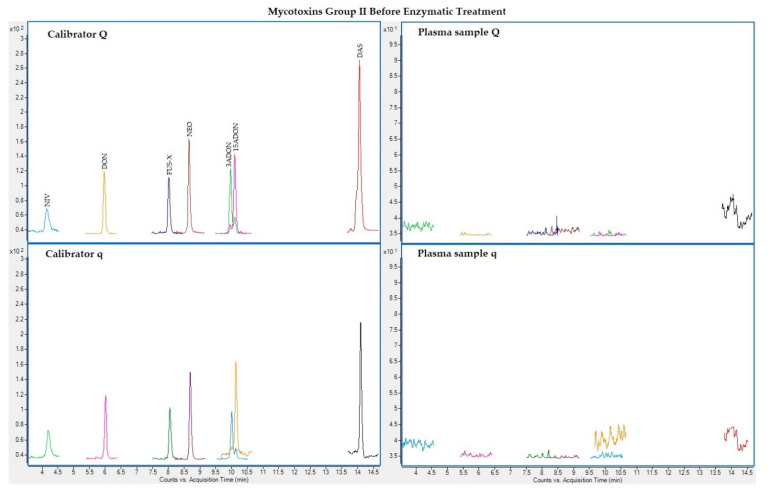
Superposed extracted chromatograms for mycotoxins group II (NIV, DON, FUS-X, NEO, 3-ADON, 15-ADON and DAS) before enzymatic treatment. Calibrator: 10xLOQ level. Plasma sample: code 1-4. Q (quantification) and q (qualification) transitions.

**Figure 3:**
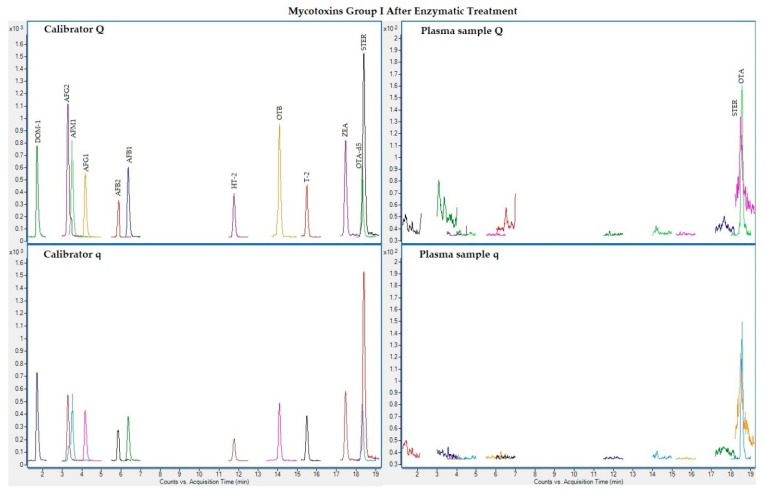
Superposed extracted chromatograms for mycotoxins group I (DOM-1, AFG2, AFM1, AFG1, AFB2, AFB1, OTB, ZEA, STER, OTA, T-2 and HT-2) after enzymatic treatment. Calibrator: 10xLOQ level. Plasma sample: code 1-4. Q (quantification) and q (qualification) transitions.

**Figure 4:**
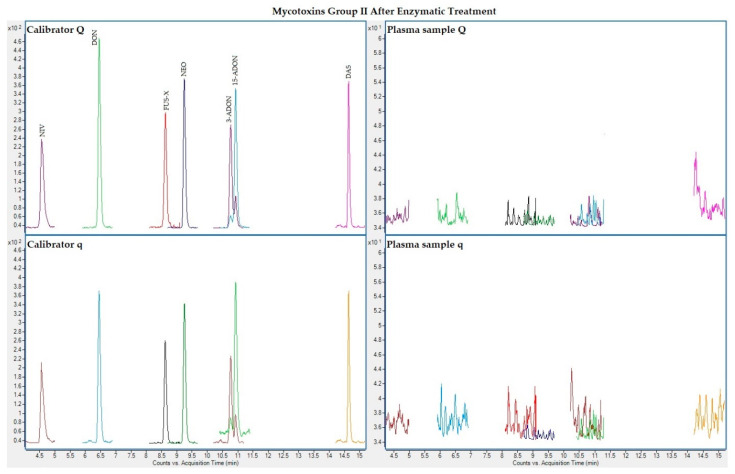
Superposed extracted chromatograms for mycotoxins group II (NIV, DON, FUS-X, NEO, 3-ADON, 15-ADON and DAS) after enzymatic treatment. Calibrator: 10xLOQ level. Plasma sample: code 1-4. Q (quantification) and q (qualification) transitions.

The following figures (Figure 1, Figure 2, Figure 3 and Figure 4) show superposed extracted chromatograms of mycotoxins in calibrators and a plasma sample (codified as sample 1–4), before and after enzymatic treatment. As it can be seen, OTA appears in samples before and after enzymatic treatment (Figure 1; Figure 3); whereas STER appears only after enzymatic treatment (Figure 3). None of the other mycotoxins analyzed have been detected in the plasma samples.

Each of the calibration curves employed in mycotoxin quantification fulfilled the criteria previously defined during method validation. Examples of the obtained calibration curves for each mycotoxin are presented in Tables S1 and S2. Identity of the compounds has been also assessed: qualification (q) and quantification (Q) transitions for each mycotoxin detected were observed in each of the individual and positive samples, the obtained relative error (RE) between q/Q ratio values for each mycotoxin in calibrators and in samples was less than 20% [32]. Additionally, retention times did not differ in samples and calibrators by more than 2.5% [33] (1.3% for OTA before and 1.6% after enzymatic treatment; 0.3% for OTB and 1.8% for STER). 

### 2.2. Plasma Samples

A total of 93 subjects, including 25 healthy controls (CNT) and 68 patients were recruited from the neurological service at the San Pedro Hospital in La Rioja (Spain). Control donors were unrelated companions of the patients with no apparent or diagnosed neurological diseases. Patients were divided by pathologies: 44 patients with PD and 24 with AD. The diagnosis and disease progression of the recruited patients was carried out by expert neurologists according to the Hoehn and Yahr (HY) scale for PD (Table S3) and to the Global Deterioration Scale (GDS) for AD (Table S4). All the AD patients already showed clinical signs of mild cognitive decline or dementia (from 3 to 7, GDS scale). The PD patients were subdivided in two groups as the disease progression was different among patients with individuals showing no impaired postural reflexes (1 and 2, HY scale) and others already showing impaired postural reflexes (from 2.5 to 3, HY scale). However, due to the low number of samples, the statistical analysis of the PD patients was carried out as a single group. 

The number of subjects enrolled for each group and sub-group is reported, together with gender, age range and disease progression, in Table 1. Complete individual information about the subjects recruited for the study are given in the supplementary materials (Tables S5 and S6). 

### 2.3. Presence of Mycotoxins

From the 19 mycotoxins evaluated, only OTA, OTB and STER were detected in the samples. OTA was present before and after the treatment with the mixture of β-glucuronidase/arylsulfatase, while OTB was only detectable before and STER appeared only after the enzymatic treatment. It should be noted that due to the limited volume of some samples, the evaluation after enzymatic treatment was possible only for 74 out of the 93 samples collected. The analytical results obtained for OTA, OTB and STER are summarized in Table 2. Due to the confirmation of identification criteria (q and Q transitions should be present and comparing with calibrators RE for both, q/Q ratio and retention time, should be < 20% or 2.5%, respectively), samples with values ≥LOD have been used for data handling analysis. Therefore, the calculations were performed adopting a lower bound substitution method [34]. 

Only OTA (77% of the samples) and OTB (13% of the samples) were detected (>LOD) in the samples processed before the enzymatic treatment. Regarding the presence of OTA, samples below the LOD were found across all the groups. The maximum level of OTA (8.81 ng/mL) corresponded to a 66 years-old woman of the control group. 

Regarding the control population, OTA and OTB were present in 92% and 24% of the samples respectively. These results are in agreement with a recent study carried out in Navarra, a neighboring region in Spain. In that study, OTA was present in 97.3% and OTB in 10% of the samples (n = 438) [35] measured with the same method as the one applied in the current study [36]. In the present study, the mean OTA levels (2.15 ng/mL) are slightly below the ones measured in Navarra (2.87 ng/mL). OTA levels ranged from LOD to 8.81 ng/mL, while in the samples from Navarra, OTA concentration ranged between LOD and 19.9 ng/mL (with a sample reaching 45.7 ng/mL in a 66-year-old woman). Regarding gender, and also in agreement with the Navarra study [35], it could be observed that mean OTA values before enzymatic treatment tended to be higher in men than in women in all groups. Although the percentage of OTA positive samples is in agreement with previous studies carried out in Spain, the mean and maximum OTA levels in the CNT population of the present study and the recent Navarra study [35] are higher than those of previous studies from Spain. As this information was obtained before 2011, our data support the need to continue biomonitoring mycotoxin levels in the general population. As previously observed [35], OTB was present in few samples and always co-occurring with OTA (except for one CNT man). Surprisingly, OTB was not detected in AD group. OTB is the dechlorinated analogue of OTA and has not been measured in the human biomonitoring studies carried out so far. OTB can be present in human samples as it is known to be present is some food matrices, such as wine [37], but might be also a human metabolite of OTA [38]. Unfortunately, in the present study it is impossible to discriminate between the possible sources of OTB or to know if the absence of OTB in the AD group could be due to a different food consumption pattern of the patients or to a different metabolism due to the disease, or if it is due to the low number of samples analyzed. 

The analysis of the data after the enzymatic treatment revealed that OTA was still detected (89% of the samples). Moreover, as previously observed in the healthy donors of Navarra [35], STER appeared in the majority of the samples (88%), while OTB was not detected anymore. 

When comparing the presence of OTA before and after the enzymatic treatment, the % of positive samples before enzymatic treatment in CNT was higher than in patients; whereas, after enzymatic treatment the presence of OTA diminished for CNT while it increased in the AD and PD patients. Some authors support the hypothesis that OTA conjugates might be formed during human metabolism [39,40,41]. However, more data are needed to understand and monitor the human metabolism of OTA. 

In this study, and again in line with the study conducted in Navarra, STER only appeared after treating the samples with β-glucuronidase/arylsulfatase and this mycotoxin was found positive (>LOD) in 88% of the analyzed samples. These data support the hypothesis that STER-glucuronides are formed during human metabolism [35,42].

### 2.4. Statistical Analysis

According to the analytical results, the statistical calculations were performed only for OTA and STER. OTB was not included in the statistical analysis because only 12 samples (13%) were ≥ LOD. The hypothesis of normality distribution (Shapiro–Wilk test) was refused, thus non-parametrical tests, which do not imply any distribution assumption, were used for the statistical treatment.

STER was detected only after enzymatic treatment, therefore, the statistical analysis for STER was performed on the 74 available results obtained after enzymatic digestion. On the other hand, for OTA two datasets of results were available, the one obtained processing data before the enzymatic treatment and the one obtained after enzymatic treatment (i.e., OTAbefore, OTAafter). Due to the incompleteness of the OTAafter dataset (74 records), it was decided to perform statistical calculations taking into account only the OTAbefore dataset (93 records).

To confirm the decision to exclude the OTAafter dataset, a Wilcoxon matched-pairs signed-ranks test was conducted to explore possible differences between paired results of the OTA before and after datasets. The test did not highlight any significant difference when applied to OTAbefore and OTAafter values of the total population group (n = 74, z = −1.438, *p*-value = 0.1503), confirming that the complete OTAbefore dataset fits for the purpose of the statistical evaluations.

As mentioned before, no previous studies monitoring mycotoxins in plasma samples from PD or AD patients are available. Therefore, with the aim of capturing all possible differences and associations of OTA and STER concentration levels between groups and sub-groups, the Wilcoxon rank-sum test or Kruskal–Wallis test for multi variable comparison was run. In addition, possible differences with statistical significance were explored dividing groups by sex, and disease (patients with PD and AD), also taking into consideration the disease stage (HY or GDS scale).

A summary of all differences, which were statistically significant (*p*-value < 0.050) when comparing OTA or STER distributions among groups, between CNT and patient group (as a whole and as PD and AD group), between sexes and among groups in each sex and sex variable, are shown in Table 3.

The differences between distribution levels of OTA were found to be statistically significant when comparing CNT group, PD and AD, but this difference (*p*-value = 0.0447) is guided by the comparison PD/AD (*p*-value = 0.0114), being PD the patient group characterized by higher values. When considering the disease stage, comparing PD sub-group 1 and PD sub-group 2 distributions, no difference was observed, indicating that in the PD group the disease stage does not influence a difference in the distribution. In the gender comparison, men and women OTA levels compared gave statistical difference either in all subjects and in patients’ group (PD + AD), with men showing always higher levels than women. Hence, the distributions of OTA are not different comparing CNT and patients’ group but it seems that the gender might act as a driver for the differences in OTA level distributions more than the diagnosis disease. Besides, in testing the differences of OTA distributions for men and women separately, among groups (CNT, PD, AD) the test did not show any difference. The statistical analysis on STER levels between the CNT and patients group showed distributions statistically different (*p*-value < 0.0001), with CNT values being lower. The CNT/PD/AD group showed statistically different distributions (*p*-value = 0.0001) and the difference was also confirmed comparing CNT/PD and CNT/AD (*p*-value < 0.0001 in both cases), with median lower value in AD group. Therefore, in STER levels the distribution of the CNT group was always different in the comparisons with patients and disease groups. Whilst comparing gender distributions, between men and women, any statistical difference was highlighted, STER levels in men and women individual groups were different in all comparisons (CNT/PD/AD, CNT/patient group, CNT/PD, CNT/AD), and men and women in CNT group were always lower than in patient groups. This suggests that the diagnosis disease may influence the STER level distributions.

Differences between concentration levels of OTA and STER were also investigated comparing the two patients’ groups (PD and AD) distributions in each sex (in men and women), and comparing the disease scale, GDS in AD patient group and HY scale in AD patient group, and no statistical difference raised. Neither OTA nor STER distribution showed any difference also comparing the HY scale as the binary variable HY_d = 0 (PD Sub-group 1, HY scale in the range 1–2) and HY_d = 1 (PD Sub-group 2, HY scale in the range 2.5–3). 

Thus, comparing OTA and STER distribution behavior, it is pinpointed that for OTA differences are inside the gender (with M > W) and inside patients’ groups (PD > AD), and a marked statistical difference came out in all comparisons. As for STER, differences in the distributions are registered comparing CNT and patients’ group (patients’ group > CNT), and all the appraisals always tested a statistical difference. On the other hand, the two mycotoxins investigated did not emphasize any difference through exploration within patients’ sub-groups and/or the disease stage.

To the best of our knowledge no other studies are available to compare mycotoxins levels in patients with neurodegenerative diseases. However, some authors have suggested the need to further evaluate the role of mycotoxins in the deterioration of the clinical manifestations of autism spectrum disorders [43,44]. Indeed, the authors highlighted statistically significant differences comparing mycotoxins (DON, DOM-1 in urine and AFM1, OTA and FB1 in plasma) between the autistic and control groups of children. The results of the present study are also in line with a more recent study by our group [45], in which OTA incidence and plasma concentrations were found to be higher in control group children than in children with autism spectrum or attention deficit hyperactivity disorders. Regarding influence of sex, and according with the results from this study, in most of the published studies plasma OTA men levels were higher than in women [46]; although Warensjö et al. (2020) in Swedish adolescents [47], and Coronel et al. (2011) [48] did not find differences in OTA plasma levels between men and women. 

Finally, to assess the relationship between OTA and STER levels in plasma and the age variable, a Spearman’s rank correlation coefficient (rho) was calculated. All the rho with statistical significance (*p*-values < 0.050) are summarized in Table 4. Generally, weak negative and positive correlations were found: OTA and STER weakly correlate with age. In particular, OTA levels decrease with age (negative correlations, rho < 0) and the rho value increases when the PD group or men’s group is selected; besides, the rho value is even higher when the values of men in the PD group is selected. Conversely, STER correlations are positive and are statistically significant when the women’s group is selected.

**Figure 5:**
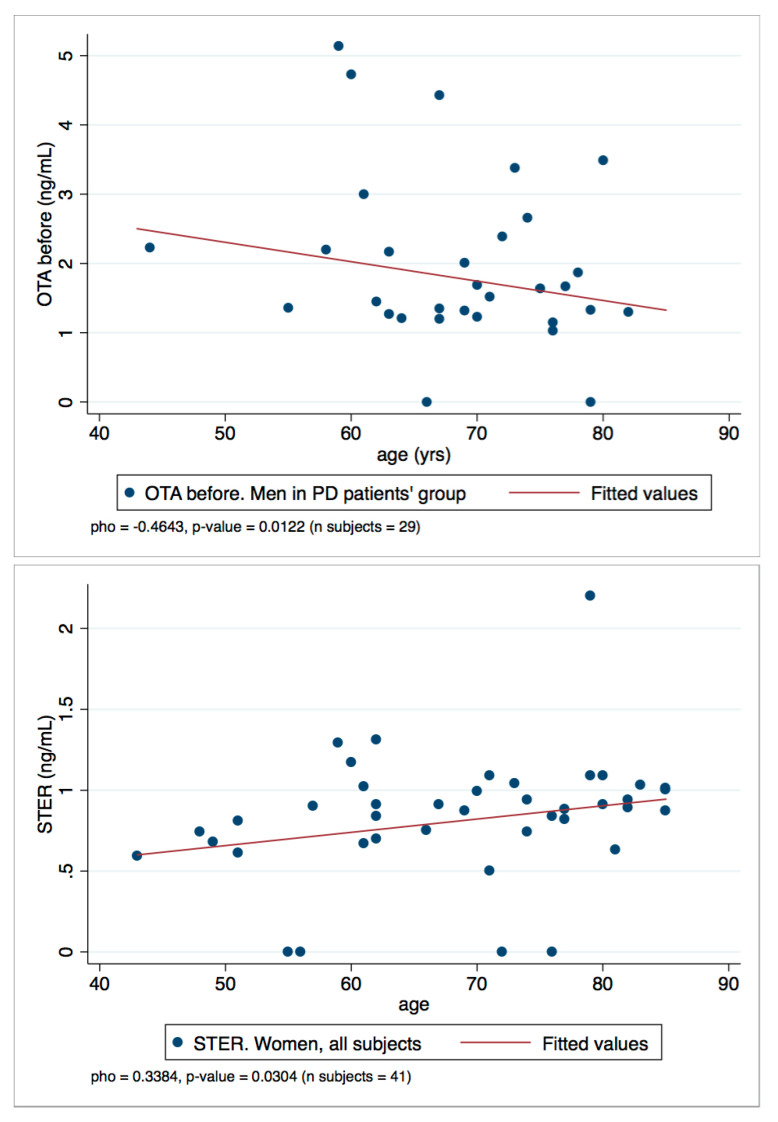
Scatter plots OTA/men in PD patient’s group and STER/women all subject.

To represent the correlations observed, the scatter plots OTA/men in PD patient’s group and STER/women all subjects are reported in Figure 5. They show the two-variables scatter plot for toxins and age, and the fitted line helps to visualize the correlation.

## 3. Conclusions

We present the results obtained in a first HBM study on the analysis of 19 mycotoxins and metabolites in plasma samples from healthy and patient (AD and PD) volunteers in La Rioja (Spain).

The results of the study suggest some differences between both control and patients in OTA and STER levels. The reason behind this difference is unknown. Several factors might be influencing the outcome observed such as differences in diets, altered metabolism due to the disease or the fact that gender and age might have an important role in the levels of mycotoxins detected in plasma. All these confounding factors should be carefully evaluated with studies including higher number of individuals. In the present study, differences in OTA between healthy companions and patients have been observed but the differences seem to be more related with gender than the disease itself. Moreover, OTA tended to decrease with age, especially in the PD group. One of the main findings is that STER appeared only after β-glucuronidase/arylsulfatase treatment, supporting the hypothesis that STER-glucuronides are formed during human metabolism. Moreover, STER plasmatic levels were found to be higher in the patients, with no differences between pathologies. Moreover, STER levels correlated positively with age in women. 

When interpreting the data, it is important to point out that the present study was not designed to explain the pathobiology of PD or AD, but rather to explore if the presence of a possible environmental risk factor may act as perturbative agents involved in the gene–environment interaction. Samples from the control group match previous data obtained in a similar population in a neighboring region of Spain and using the same LC/MS-MS analysis [35]. However, when comparing healthy individuals between both studies it should be noted that: (i) the age range is different, as the majority of the samples in the present study were obtained from people older than 60 years, especially in the AD and PD groups, as expected for age-related diseases; and (ii) the number of control samples which is low in the present study.

Moreover, other limitations should be also taken into account when interpreting data from the present study; many of the data were very close to the LOD and the number of samples were limited (especially in AD men). Overall, our results show some statistically significant differences between the control group and patients of neurodegenerative diseases but also point to some age- and sex-related responses that could be in turn be influencing the metabolism. 

## 4. Materials and Methods

### 4.1. Reagents

LC-MS grade methanol (Honeywell Riedel-de-Haën, Seelze, Germany), LC grade acetonitrile (ACN) (Merck, Darmstadt, Germany), MS grade formic acid (purity > 98%), ammonium formate (both from Fluka Sigma-Aldrich, Mannheim, Germany), and deionized water (>18 MΩ cm^−1^ resistivity) purified in an Ultramatic Type I system (Wasserlab, Navarra, Spain) were used for sample preparation and LC-MS/MS analysis. Captiva EMR-lipid (3 mL) cartridges were obtained from Agilent Technologies (Santa Clara, CA, USA). 

All mycotoxins (reference material, purity ≥ 98%) were purchased from Sigma-Aldrich (St. Louis, MO, USA) as ACN solutions at the following concentrations AFM1, AFG2 and AFB2: 0.5 μg/mL; AFG1 and AFB1: 2 μg/mL; OTA-d5 and OTB: 10 μg/mL; STER and DOM-1: 50 μg/mL; NIV, DON, 3-ADON, 15-ADON, NEO, DAS, FUS-X, ZEA and T-2 and HT-2 toxins: 100 μg/mL. The reference materials were stored at −20 °C.

Standard stock solutions were prepared in ACN and stored at −20 °C. The working solution, that includes all the mycotoxins, was prepared by diluting the corresponding individual stock solutions in ACN. Stability of the standard and working stock solutions in this storage conditions were previously assessed [36].

### 4.2. Subjects

Informed written consent was obtained from all participants prior to study inclusion. A total of 94 subjects, including 44 patients with mild PD, 25 patients with dementia not related with PD and 25 healthy controls, were recruited from the neurological service at the San Pedro Hospital in La Rioja (Spain) with ethical approval (“Study of lipidic profiles in serum/plasma samples from Parkinson´s disease patients for the obtention a differential clinical pattern for diagnosis purposes” Ref: CEICLAR PI- 212, approved 4 April 2016) from the local ethical committee on human experimentation (CEImLar, Comité Ética de Investigación con medicamentos de La Rioja). Patients were diagnosed by experienced neurologists. PD patients were assessed by the modified HY scale (Table S3) while patients with dementia not related to PD were assessed by the GDS (Table S4) to determine their disease stage. Healthy controls included spouses or unrelated companions of patients with no apparent or known neurological disease or comorbidities. 

### 4.3. Plasma Collection

Blood samples were obtained at a medical visit. Blood was collected into 4 mL EDTA-coated tubes (Vacutainer, ref # 368171) and the plasma was separated within 2 h by centrifugation at 2200× *g* for 15 min at room temperature. Plasma was removed immediately and transferred into coded vials in 0.2 mL aliquots to avoid repeated freeze/thaw cycles and then stored at −80 °C. Appropriate care was taken to avoid contamination of the plasma samples with cells or components of the pellet obtained from the centrifugation. 

### 4.4. Sample Preparation

Plasma treatment before and after enzymatic hydrolysis was as described in Arce-López et al. (2020) [35,36]. Concisely, the plasma samples (400 µL) were added to a Captiva EMR-lipid cartridge, which contained 1200 µL of acidified ACN (1% formic acid). Vacuum was applied, and after 5 min, two 400 µL aliquots (one for each of the mycotoxin groups that will be investigated) from the effluent were separated and then evaporated to dryness (60 °C). This methodology was used for the detection of 19 compounds (mycotoxins and metabolites), classified in two groups (according to the different elution program needed for the chromatographic separation): DOM-1, AFG2, AFM1, AFG1, AFB2, AFB1, OTB, ZEA, STER, OTA, T-2, HT-2 (group I) and NIV, DON, FUS-X, NEO, 3-ADON, 15-ADON and DAS (group II). The residue was reconstituted with 200 μL of mobile phase in the proportion corresponding to the intended group of mycotoxins to be analyzed (40% B for group I and 5% B for group II). Prior to chromatographic analysis, the solution was vortexed (5 min) and filtered (PVDF, 0.45 μm, Merck Millipore, Ireland). The procedure for enzymatic hydrolysis was: 400 µL of plasma were treated with 50 µL of a mixture of glucuronidase/sulfatase enzymes (250 U/mL, 0.2 U/mL in PBS; from Helix Pomatia (Sigma Aldrich, Mannheim, Germany). After agitation, samples were incubated overnight (37 °C) in a water bath. Then, plasma sample clean-up, using Captiva-EMR cartridges, was carried-out in a similar manner to that described above. 

### 4.5. LC/MS-MS Analysis

LC-MS/MS analysis were performed in an LC system 1200 series coupled to a 6410 Triple Quadrupole (QqQ) in ESI(+) mode, both from Agilent Technologies (Mannheim, Germany). Separation was carried out at 45 °C on an Ascentis Express C18, 2.7 μm particle size 150 × 2.1 mm column (Supelco Analytical, St. Louis, MO, USA) with mobile phase composed of 5 mM ammonium formate and 0.1% formic acid in water (A) and 5 mM ammonium formate and 0.1% formic acid in a 95:5 methanol/water (B) in gradient conditions. The injection volume was 20 μL and the gradient elution was carried out at the flow rate of 0.4 mL/min. Data acquisition parameters are described in Arce-López et al. (2020) [36].

Samples were analyzed grouped in analytical sequences. Each sequence included, along with the samples, eight matrix-matched calibrators. These calibrators were employed in the preparation of calibration curves, which served for the quantification of mycotoxins in the samples analyzed in the same sequence. The acceptance criteria for the calibration curves were: minimum of six points, a determination coefficient (R^2^) > 0.99 and back-calculated concentration for each calibrator not differing (expressed as RE) from the nominal value by more than 15% (20% for LOQ level) [32]. In addition, retention times should not differ by more than 2.5% between samples and calibrators [33].

Depending on the volume of plasma available, not all the samples could be analyzed after enzymatic treatment. 

### 4.6. Analytical Method Validation

Validation of both procedures (before and after enzymatic hydrolysis) following FDA and EU guidelines were described in Arce-Lopez et al. (2020) [35,36]. LOD values were: 1.35 ng/mL for DOM-1; 0.35 ng/mL for AFG2, 0.18 ng/mL for AFM1; 0.07 ng/mL for AFG1 and AFB2; 0.04 ng/mL for AFB1; 2.70 ng/mL for HT-2; 0.40 ng/mL for OTA and OTB; 0.20 ng/mL for T-2 and STER; 1.80 ng/mL for ZEA; 9.10 ng/mL for NIV; 1.94 ng/mL for DON; 1.95 ng/mL for FUS-X; 0.18 ng/mL for NEO; 0.70 ng/mL for 3-ADON; 1.20 ng/mL for 15-ADON and 0.15 ng/mL for DAS. Recovery values (in intermediate precision conditions) before enzymatic treatment were from 68.8% for STER to 97.6% for DAS (RDS ≤ 15% for all the mycotoxins) and no differences were found after enzymatic treatment. Matrix effects were also evaluated before and after enzymatic treatment, and there were not differences in most of the mycotoxins, obtaining RDS values ≤ 15% for all of them. Stability after two freeze–thaw cycles was assessed at two concentration levels (6 and 30xLOQ) (three replicates per level). All mycotoxins were stable (RSD < 15%) (Table S7).

### 4.7. Statistical Analysis and Data Handling

When treating analytical data with statistic, it is important to elucidate the kind of distribution that characterized the dataset. In fact, the descriptors and package tests to be used are different if we have normal distributed or non-normal distributed data. Shapiro–Wilk test was used to verify the normal distribution of the mycotoxin concentration levels investigated. As the hypothesis of normality was refused, a non-parametrical (which do not imply any distribution assumption) set of tests was used for the statistical treatment.

In order to assess the possible differences between concentration levels of OTA and STER in groups and sub-groups, the Wilcoxon rank-sum test (Mann–Whitney two-sample statistic) was used [49].

In statistics, the Wilcoxon rank-sum test is a non-parametric test used to test whether two samples are likely to derive from the same population verifying that for two randomly selected values X and Y from the two populations, the probability of *X* being greater than *Y* is equal to the probability of *Y* being greater than *X*. For the same purpose, a Kruskal–Wallis test was used where the collation of variables implied more than one distribution [50].

To assess the correlation between mycotoxin levels and age (quantitative variable) the Spearman’s rank correlation coefficient (or Spearman’s rho) was used. 

All tests were conducted with a level of significance of 5%. The outputs are reported together with the level of statistical significance, *p*-value. All the analyses were conducted by means of STATA14 software (Stata/IC 14.0, Copyright 1985–2015 StataCorp LP).

As regards the data handling for the setting of the data set, quantitative and qualitative variables were treated as follows:

OTA and STER level. Quantitative variable, non-negative variable expressed as values in ng/mL. Values were assigned as follows:

Value = 0 ng/mL; when analytical result derived from the analysis was found <LOD, thus no concentration could be derived (substitution method with Lower Bound approach). LOD value was defined during validation.

Value = ng/mL; to those values found ≥ LOD. Despite the lower precision of the data (RSD_r_ > 20%), samples ≥ LOD could be used due to the confirmation of identification criteria established during method validation.

Values = numerical value of OTA and STER. Analytical results in ng/mL were rounded to the second figure after the comma.

Age. Quantitative variable, non-negative variable expressed as years. This variable has been set defining the age at the moment of the sampling. 

Sex. Dicotomic variable: men, women.

HY scale. A quantitative variable used to scale diagnosis of PD patients (Table S3). In addition, a binary variable (HY_d = 0 and HY_d = 1) was defined: HY_d = 0 for PD patients who scored HY scale in the range 1–2 (not impaired postural reflexes); HY_d = 1 for PD patients who scored HY scale in the range 2.5–3 (impaired postural reflexes).

GDS scale. A quantitative variable used to scale diagnosis of patience with AD (Table S4).

## Figures and Tables

**Table 1:** Number of subjects (men/women) recruited for each group and sub-group.

Groups/ Sub Groups	Disease Stage	N° of Subjects (M/W *)	Age Range (Years)
Subjects		93 (43/50)	43–85
Control (CNT)		25 (8/17)	43–81
Patients		68 (35/33)	44–85
PD		44 (29/15)	44–85
PD sub-group 1.	HY scale: 1–2	35 (23/12)	44–85
PD sub-group 2	HY scale: 2.5–3	9 (6/3)	62–83
AD	GDS scale 3–7	24 (6/18)	60–85

* M = men W = women.

**Table 2:** Summary of mycotoxins levels before and after enzymatic treatment stratified by diagnosis.

	Before Enzymatic Treatment		After Enzymatic Treatment	
	OTA	OTB	OTA	STER
**Total population**				
Positive samples (N/%)	72 (77%)	12 (13%)	66 (89%)	65 (88%)
Mean value (M/W); ng/mL	1.77 (2.10/1.50)	0.07 (0.10/0.04)	2.00 (1.86/2.10)	0.81 (0.78/0.84)
Median (M/W); ng/mL	1.65 (1.92/1.47)	0 (0/0)	1.68 (1.52/1.93)	0.87 (0.86/0.88)
Range (min-max); ng/mL	0–8.81	0–0.75	0–6.45	0–2.20
**Control (CNT)**				
Positive samples (N/%)	23 (92%)	6 (24%)	19 (86%)	16 (73%)
Mean value (M/W); ng/mL	2.15 (2.47/1.99)	0.12 (0.22/0.07)	2.51 (2.11/2.69)	0.51 (0.53/0.51)
Median (M/W); ng/mL	1.56 (2.19/1.50)	0 (0.22/0)	2.35 (2.01/2.41)	0.65 (0.69/0.63)
Range (min-max); ng/mL	0–8.81	0–0.75	0–6.25	0–0.90
**Patients**				
Positive samples (N/%)	49 (72%)	6 (9%)	47 (90%)	49 (94%)
Mean value (M/W); ng/mL	1.64 (2.01/1.24)	0.05 (0.07/0.02)	1.78 (1.79/1.76)	0.94 (0.84/1.02)
Median (M/W); ng/mL	1.66 (1.92/1.45)	0 (0/0)	1.46 (1.46/1.43)	0.94 (0.87/0.97)
Range (min-max); ng/mL	0–6.44	0–0.71	0–6.45	0–2.22
***Patients with PD***				
Positive samples (N/%)	33 (75%)	6 (14%)	31 (91%)	31 (91%)
Mean value (M/W); ng/mL	1.93 (2.13/1.54)	0.07 (0.09/0.05)	1.88 (1.82/1.99)	0.90 (0.82/1.04)
Median (M/W); ng/mL	1.86 (1.98/1.50)	0 (0/0)	1.53 (1.41/1.61)	0.95 (0.87/1.02)
Range (min-max); ng/mL	0–6.44	0–0.71	0–6.45	0–1.31
***Patients with AD***				
Positive samples (N/%)	16 (67%)	0 (0%)	16 (89%)	18 (100%)
Mean value (M/W); ng/mL	1.11 (1.47/0.99)	0 (0/0)	1.59 (1.64/1.57)	1.01 (0.98/1.02)
Median (M/W); ng/mL	1.46 (1.75/1.45)	0 (0/0)	1.39 (1.96/1.37)	0.91 (0.92/0.91)
Range (min-max); ng/mL	0–2.11	0	0–3.94	0.74–2.20

M = men W = women.

**Table 3:** Summary of statistically significant differences in OTA/STER distribution concentrations (ng/mL) among groups, between CNT, PD and AD, between sexes and among groups in each sex. The obtained *p*-values are reported.

	OTA	STER
CNT/patient group (PD + AD)	-	<0.0001
CNT/PD/AD	0.0447	0.0001
CNT/PD	-	<0.0001
CNT/AD	-	<0.0001
PD/AD	0.0114	-
M/W	0.0014	-
M/W in patient group (PD + AD)	0.0013	-
M/W in patient group PD	*(0.0613)*	0.0304
Sex = M in CNT/PD/AD	-	0.0128
Sex = M in CNT/patient group (PD + AD)	-	0.0047
Sex = M in CNT/PD	-	0.0105
Sex = M in CNT/AD	-	0.0080
Sex = W in CNT/PD/AD	-	0.0001
Sex = W in CNT/patient group (PD + AD)	-	0.0001
Sex = W in CNT/PD	-	<0.0001
Sex = W in CNT/AD	-	<0.0001

M = men W = women.

**Table 4:** Spearman correlations (rho values, *p*-values and number of observations).

	OTA			STER		
	rho	*p*-Value	N° Observations	rho	*p*-Value	N° Observations
Age	−0.2033	0.0507	93	0.2686	0.0207	74
Age in *PD*	−0.3304	0.0285	44	-	-	-
Age in men	−0.3775	0.0126	43	-	-	-
Age in women	-	-	-	0.3384	0.0305	41
Age in PD men	−0.4643	0.0122	29	-	-	-

## Data Availability

Data is contained within the article or supplementary material.

## Supplementary Materials

The following are available online at https://www.mdpi.com/article/10.3390/toxins13070477/s1, Tables S1 and S2: Examples of calibration curves. Table S3: Hoehn and Yahr scale used for the diagnosis of patients with Parkinson´s Disease. Table S4: Global Deterioration Scale (GDS) used for the diagnosis of patients with Alzheimer. Table S5: Levels of mycotoxins in men. Table S6: Levels of mycotoxins in women Table S7. Stability at two concentration levels (6xLOQ and 30xLOQ) after two freeze–thaw cycles.

## Abbreviations

15-ADON	15-acetyldeoxynivalenol
3-ADON	3-acetyldeoxynivalenol
Aβ	amyloid beta
ACN	acetonitrile
AD	Alzheimer´s disease
AFB1	aflatoxin B1
AFB2	aflatoxin B2
AFG1	aflatoxin G1
AFG2	aflatoxin G2
AFM1	aflatoxin M1
CIRS	chronic inflammatory response syndrome
CNT	control
DAS	diacetoxyscirpenol
DOM-1	deepoxy-deoxynivalenol
DON	deoxynivalenol
FUS-X	fusarenon-X
GDS	Global Deterioration Scale
HBM	human biomonitoring
HY	Hoehn and Yahr
LC	liquid chromatography
LC-MS/MS	liquid chromatography coupled to mass spectrometry in tandem
LOD	limit of detection
LOQ	limit of quantification
NEO	neosolaniol
NIV	nivalenol
OTA	ochratoxin A
OTA-d_5_	ochratoxin A-(*phenyl*-d_5_)
OTB	ochratoxin B
PD	Parkinson´s disease
q	transition of qualification
Q	transition of quantification
QqQ	triple quadrupole
RE	relative error of the mean
SD	standard deviation
STER	sterigmatocystin
ZEA	zearalenone
