# Supplementary material for: Biomonitoring of Mycotoxins in Plasma of Patients with Alzheimer’s and Parkinson’s Disease
Source: Toxins (Basel). 2021 Jul 10;13(7):477. doi: 10.3390/toxins13070477 (PMC8310068; doi:10.3390/toxins13070477)
Supplement: Supplementary file 1 [file toxins-13-00477-s001.zip › toxins-1266100 supplementary ready 2.pdf]

Supplementary Materials: Biomonitoring of Mycotoxins in Plasma of Patients With Alzheimer's and Parkinson's Disease

Beatriz Arce-López, Lydia Alvarez-Erviti, Barbara De Santis, María Izco, Silvia López-Calvo, Maria Eugenia Marzo-Sola, Francesca Debegnach, Elena Lizarraga, Adela López de Cerain, Elena González-Peñas and Ariane Vettorazzi

Table S1. Calibration curves for each of the analysed mycotoxins before enzymatic treatment.

Mycotoxin	Range (ng/mL)	LOD (ng/mL)	R^2	Calibration curve	Weight
DOM-1	6.0-180.0	1.35	0.9995	Y = 15.74x + 6.46	None
AFG2	0.8-24.0	0.35	0.9960	Y = 210.18x + 35.09	1/x
AFM1	0.8-24.0	0.18	0.9957	Y = 152.96x - 14.03	1/x
AFG1	0.3-9.0	0.07	0.9937	Y = 444.18x - 28.91	None
AFB2	0.3-9.0	0.07	0.9993	Y = 304.58x + 17.35	None
AFB1	0.2-6.0	0.04	0.9958	Y = 664.76x - 17.77	1/x
HT-2	6.0-180.0	2.70	0.9956	Y = 20.35x + 2.85	1/x
OTB	1.0-30.0	0.40	0.9956	Y = 287.06x – 52.71	1/x
T-2	1.0-30.0	0.20	0.9956	Y = 864.36x – 82.29	1/x
ZEA	4.0-120.0	1.80	0.9962	Y = 44.09x + 31.86	1/x
OTA-d_5	2.0-60.0	0.40	0.9976	Y = 95.89x – 79.54	1/x
STER	1.0-30.0	0.20	0.9948	Y = 172.06x – 5.30	1/x
NIV	20.4-612.0	9.10	0.9991	Y = 1.24x – 8.37	None
DON	8.72-361.6	1.94	0.9967	Y = 4.53x + 8.64	None
FUS-X	7.0-210.0	1.95	0.9963	Y = 4.43x – 3.33	None
NEO	0.8-24.0	0.18	0.9960	Y = 60.08x + 37.78	None
3-ADON	1.75-52.5	0.70	0.9967	Y = 22.91x + 9.94	None
15-ADON	2.72-81.6	1.20	0.9949	Y = 18.84x – 9.39	None
DAS	0.7-21.0	0.15	0.9949	Y = 62.30x + 898.95	None

LOD: limit of detection.

Table S2. Calibration curves for each of the analysed mycotoxins after enzymatic treatment.

Mycotoxin	Range (ng/mL)	LOD (ng/mL)	R^2	Calibration curve	Weight
DOM-1	6.0-180.0	1.35	0.9974	Y = 49.54x – 217.53	None
AFG2	0.8-24.0	0.35	0.9961	Y = 683.65x - 294.01	None
AFM1	0.8-24.0	0.18	0.9937	Y = 349.85x - 91.50	None
AFG1	0.3-9.0	0.07	0.9945	y = 946.42x - 101.23	None
AFB2	0.3-9.0	0.07	0.9923	y = 950.98x - 136.14	None
AFB1	0.2-6.0	0.04	0.9915	y = 1712.34x - 167.77	None
HT-2	6.0-180.0	2.70	0.9954	y = 32.16x – 101.54	None
OTB	1.0-30.0	0.40	0.9949	y = 394.87x – 138.17	None
T-2	1.0-30.0	0.20	0.9951	y = 192.83x – 150.37	None
ZEA	4.0-120.0	1.80	0.9920	y = 59.48x – 175.70	None
OTA-d_5	2.0-60.0	0.40	0.9937	y = 129.73x – 65.49	None
STER	1.0-30.0	0.20	0.9957	y = 421.69x – 232.27	1/x
NIV	20.4-612.0	9.10	0.9945	y = 3.20x – 16.37	None
DON	8.72-361.6	1.94	0.9968	y = 12.22x – 22.80	None
FUS-X	7.0-210.0	1.95	0.9991	y = 7.29x – 10.09	None
NEO	0.8-24.0	0.18	0.9981	y = 105.19x – 39.67	None
3-ADON	1.75-52.5	0.70	0.9948	y = 42.32x – 17.01	None
15-ADON	2.72-81.6	1.20	0.9908	y = 35.39x – 52.29	None
DAS	0.7-21.0	0.15	0.9952	y = 148.97x – 53.80	None

LOD: limit of detection.

Table S3. Hoehn and Yahr scale used for the diagnosis of patients with Parkinson´s Disease.

Level	Clinical Characteristics
1.0	Unilateral involvement only
1.5	Unilateral and axial involvement
2.0	Bilateral involvement without impairment of balance
2.5	Mild bilateral disease with recovery on pull test
3.0	Mild to moderate bilateral disease; some postural instability; physically independent
4.0	Severe disability; still able to walk or stand unassisted
5.0	Wheelchair bound or bedridden unless aided

Table S4. Global Deterioration Scale (GDS) used for the diagnosis of patients with Alzheimer´s disease.

Stages	Level	Clinical Characteristics
Pre-dementia	1<br>No cognitive decline	No subjective complaints of memory deficit. No memory deficit evident on clinical interview.
	2<br>Very mild cognitive decline (Age Associated Memory Impairment)	Subjective complaints of memory deficit, most frequently in following areas:<br>(a) forgetting where one has placed familiar objects;<br>(b) forgetting names one formerly knew well.<br>No objective evidence of memory deficit on clinical interview. No objective deficits in employment or social situations. Appropriate concern with respect to symptomatology.
	3<br>Mild cognitive decline (Mild Cognitive Impairment)	Earliest clear-cut deficits.<br>Manifestations in more than one of the following areas:<br>(a) patient may have gotten lost when traveling to an unfamiliar location;<br>(b) coworkers become aware of patient's relatively poor performance; (c) word and name finding deficit becomes evident to intimates;<br>(d) patient may read a passage or a book and retain relatively little material;<br>(e) patient may demonstrate decreased facility in remembering names upon introduction to new people;<br>(f) patient may have lost or misplaced an object of value;<br>(g) concentration deficit may be evident on clinical testing.<br>Objective evidence of memory deficit obtained only with an intensive interview. Decreased performance in demanding employment and social settings. Denial begins to become manifest in patient. Mild to moderate anxiety accompanies symptoms.
Dementia	4<br>Moderate cognitive decline (Mild Dementia)	Clear-cut deficit on careful clinical interview.<br>Deficit manifest in following areas:<br>(a) decreased knowledge of current and recent events;<br>(b) may exhibit some deficit in memory of ones personal history;<br>(c) concentration deficit elicited on serial subtractions;<br>(d) decreased ability to travel, handle finances, etc.<br>Frequently no deficit in following areas:<br>(a) orientation to time and place;<br>(b) recognition of familiar persons and faces;<br>(c) ability to travel to familiar locations.<br>Inability to perform complex tasks. Denial is dominant defense mechanism. Flattening of affect and withdrawal from challenging situations frequently occur.
	5<br>Moderately severe cognitive decline (Moderate Dementia)	Patient can no longer survive without some assistance. Patient is unable during interview to recall a major relevant aspect of their current lives, e.g., an address or telephone number of many years, the names of close family members (such as grandchildren), the name of the high school or college from which they graduated. Frequently some disorientation to time (date, day of week, season, etc.) or to place. An educated person may have difficulty counting back from 40 by 4s or from 20 by 2s. Persons at this stage retain knowledge of many major facts regarding themselves and others. They invariably know their own names and generally know their spouses' and children's names. They require no assistance with toileting and eating, but may have some difficulty choosing the proper clothing to wear.
Dementia	6<br>Severe cognitive decline (Moderately Severe Dementia)	May occasionally forget the name of the spouse upon whom they are entirely dependent for survival. Will be largely unaware of all recent events and experiences in their lives. Retain some knowledge of their past lives but this is very sketchy. Generally unaware of their surroundings, the year, the season, etc. May have difficulty counting from 10, both backward and, sometimes, forward. Will require some assistance with activities of daily living, e.g., may become incontinent,

Stages	Level	Clinical Characteristics
		will require travel assistance but occasionally will be able to travel to familiar locations. Diurnal rhythm frequently disturbed. Almost always recall their own name. Frequently continue to be able to distinguish familiar from unfamiliar persons in their environment. Personality and emotional changes occur.<br>These are quite variable and include:<br>(a) delusional behavior, e.g., patients may accuse their spouse of being an impostor, may talk to imaginary figures in the environment, or to their own reflection in the mirror;<br>(b) obsessive symptoms, e.g., person may continually repeat simple cleaning activities;<br>(c) anxiety symptoms, agitation, and even previously nonexistent violent behavior may occur;<br>(d) cognitive abulla, i.e., loss of willpower because an individual cannot carry a thought long enough to determine a purposeful course of action.
	7<br>Very severe cognitive decline<br>(Severe Dementia)	All verbal abilities are lost over the course of this stage. Frequently there is no speech at all -only unintelligible utterances and rare emergence of seemingly forgotten words and phrases. Incontinent of urine, requires assistance toileting and feeding. Basic psychomotor skills, e.g., ability to walk, are lost with the progression of this stage. The brain appears to no longer be able to tell the body what to do. Generalized rigidity and developmental neurologic reflexes are frequently present.

Table S5. Levels of OTA, OTB and STER (ng/mL) found in men plasma samples.

**Sample code**	**Age**	**Disease stage**[a]	**Enzymatic treatment**[b]				**Sample code**	**Age**	**Disease stage**[a]	**Enzymatic treatment**[b]				**Sample code**	**Age**	**Disease stage**[a]	**Enzymatic treatment**[b]			
			**Before**		**After**					**Before**		**After**					**Before**		**After**	
			**OTA**	**OTB**	**OTA**	**STER**				**OTA**	**OTB**	**OTA**	**STER**				**OTA**	**OTB**	**OTA**	**STER**
		CNT													***PD***					
**1-2**	69	CNT	*1.48*		2.01		**2-1**	73	2	*1.22*		n.e.	n.e.	2-26	62	1.5	*1.67*		n.e.	n.e.
**1-5**	69	CNT	3.74	*0.45*	n.e.	n.e.	**2-2**	58	2	*1.53*		2.20	1.01	2-28	79	3	*1.65*		*1.33*	1.03
**1-6**	80	CNT	4.87		3.49	*0.69*	**2-3**	69	1			*1.32*	1.23	2-30	78	1	*1.70*		*1.87*	*0.95*
**1-13**	65	CNT	*1.56*			*0.55*	**2-5**	68	2	5.92	*0.53*	n.e.	n.e.	2-31	55	1	*1.89*		*1.36*	*0.81*
**1-16**	66	CNT	*1.50*	*0.44*			**2-10**	70	1	2.39		*1.23*	1.29	2-33	64	2	*1.98*		*1.21*	*0.86*
**1-23**	73	CNT	2.82		3.38	*0.77*	**2-13**	82	1	*1.84*		*1.30*	*0.87*	2-34	63	1	2.43		2.17	*0.86*
**1-24**	60	CNT	3.75	*0.46*	4.73	*0.83*	**2-15**	61	1	2.65		3.00	*0.87*	2-36	77	3	2.17		*1.67*	1.01
**1-25**	76	CNT		*0.43*	*1.15*	*0.84*	**2-17**	59	1	4.08	*0.44*	5.14	1.03	2-37	46	1	2.50		n.e.	n.e.
		AD					**2-18**	65	1	2.25	*0.44*			2-39	63	3	*1.34*		*1.27*	1.05
**4-2**	68	3	*1.66*		n.e.	n.e.	**2-19**	60	2	2.10	*0.49*	n.e.	n.e.	2-40	67	2.5	6.44	*0.60*	4.43	1.02
**4-5**	*79*	6	*1.35*			*0.96*	**2-20**	67	1	*1.92*		*1.35*		2-42	70	2	*1.87*		*1.69*	*0.95*
**4-8**	71	7	*1.84*		*1.52*	*0.86*	**2-22**	80	1.5			n.e.	n.e.	2-43	67	2			1.20	*0.79*
**4-9**	74	6	*1.95*		2.66	1.22	**2-23**	62	3	2.56		*1.45*		2-44	44	1	2.57		2.23	*0.86*
**4-10**	81	5			n.e.	n.e.	**2-24**	76	3			*1.03*	*0.84*	2-45	75	1	*1.99*		*1.64*	*0.75*
**4-11**	72	5	*1.97*		2.39	*0.87*	**2-25**	55	1.5	2.99		n.e.	n.e.							

[a] In relation to the disease stage, Alzheimer's disease (AD) group was diagnosed based on GDS scale and the Parkinson's disease (PD) group based on HY scale. [b] Number in italics: <LOQ (OTA 2 ng/mL, OTB and STER 1 ng/mL). Empty spaces: values <LOD. LODs: OTA and OTB 0.4 ng/mL, STER 0.2 ng/mL. n.e.: no enzymatic treatment. CNT: control group.

Table S6. Levels of OTA, OTB and STER (ng/mL) found in women plasma samples.

**Sample code**	**Age**	**Disease stage**[a]	**Enzymatic treatment**[b]				**Sample code**	**Age**	**Disease stage**[a]	**Enzymatic treatment**[b]				**Sample code**	**Age**	**Disease stage**[a]	**Enzymatic treatment**[b]			
			**Before**		**After**					**Before**		**After**					**Before**			**After**
			**OTA**	**OTB**	**OTA**	**STER**				**OTA**	**OTB**	**OTA**	**STER**				**OTA**	**OTB**	**OTA**	**STER**
		CNT								***PD***									***AD***	
**1-1**	62	CNT	*0.95*		2.19	*0.70*	**2-4**	74	2			*1.46*	*0.94*	**4-1**	69	4	*1.69*		*1.23*	*0.87*
**1-3**	43	CNT	*1.82*		2.42	*0.59*	**2-6**	70	1	6.11	*0.71*	6.45	0.99	**4-3**	77	4			*1.06*	*0.88*
**1-4**	66	CNT	8.81		6.25	*0.75*	**2-7**	80	2.5			*1.91*	1.09	**4-4**	85	4	*1.88*		*1.93*	1.01
**1-7**	49	CNT	*1.46*		2.41	*0.68*	**2-8**	85	1				1.00	**4-7**	85	3	*1.53*		*1.32*	*0.87*
**1-8**	76	CNT	*1.07*		2.07		**2-9**	57	1	*1.56*		n.e	n.e	**4-13**	79	5	2.11		2.70	1.09
**1-9**	55	CNT	*1.00*				**2-11**	59	1	*1.65*		2.07	1.29	**4-14**	67	7	*1.45*		*1.72*	*0.91*
**1-10**	72	CNT	*0.76*		2.29		**2-12**	83	3	4.31		2.37	1.03	**4-15**	62	3			*1.39*	*0.91*
**1-11**	56	CNT	*1.79*		2.28		**2-14**	64	1			n.e	n.e	**4-16**	62	5			n.e	n.e
**1-12**	71	CNT	*1.50*		n.e	n.e	**2-16**	76	1.5			1.36	*0.84*	**4-18**	62	3	*1.82*			*0.84*
**1-14**	71	CNT	2.49		2.43	*0.50*	**2-21**	81	1			n.e	n.e	**4-19**	82	5	*1.51*		n.e	n.e
**1-15**	51	CNT	3.68		4.94	*0.81*	**2-27**	82	1.5	4.03		3.71	*0.89*	**4-20**	83	3			n.e	n.e
**1-17**	64	CNT	*1.33*				**2-32**	73	1	*1.82*			1.04	**4-22**	61	6			*1.17*	1.02
**1-18**	57	CNT	*1.69*		2.58	*0.90*	**2-35**	62	1	2.16		1.59	1.31	**4-23**	77	4	*1.56*		*1.53*	*0.82*
**1-19**	81	CNT	*1.64*	*0.47*	2.18	*0.63*	**2-38**	71	3			1.30	1.09	**4-24**	74	5			*1.35*	*0.74*
**1-20**	48	CNT			2.80	*0.74*	**4-17***	80	2	*1.50*		1.62	*0.91*	**4-25**	79	4			*1.39*	2.20
**1-21**	51	CNT	*1.24*		2.27	*0.61*								**4-26**	82	4	*1.47*		*1.27*	*0.94*
**1-22**	61	CNT	2.68	*0.75*	3.28	*0.67*								**4-27**	60	4	*1.39*		3.94	1.17
														**4-28**	72	5	*1.45*		n.e	n.e

[a] In relation to the disease stage, Alzheimer's disease (AD) group was diagnosed based on GDS scale and the Parkinson's disease (PD) group based on HY scale. [b] Number in italics: <LOQ (OTA 2 ng/mL, OTB and STER 1 ng/mL). Empty spaces: values <LOD. LODs: OTA and OTB 0.4 ng/mL, STER 0.2 ng/mL. n.e.: no enzymatic treatment. CNT: control group. *Incorrectly labeled sample. .

.

Table S7. Stability at two concentration levels (6xLOQ and 30xLOQ) after two freeze-thaw cycles.

**6xLOQ**	**DOM-1**	**AFG2**	**AFM1**	**AFG1**	**AFB2**	**AFB1**	**HT-2**	**OTB**	**T-2**	**ZEA**
	36.06	3.38	3.51	1.23	1.53	0.94	35.32	6.05	6.06	24.80
Ccal (ng/mL)	38.50	3.58	3.51	1.17	1.44	0.94	38.05	6.60	6.50	*
	35.02	3.29	3.66	1.23	1.53	0.93	35.28	6.28	6.07	21.95
Mean	36.53	3.42	3.56	1.21	1.50	0.94	36.21	6.31	6.21	23.37
RE (%)	1.46	28.79	25.85	32.84	16.57	22.01	0.60	5.15	3.46	2.62
RSD (%)	4.89	4.43	2.46	2.62	3.63	0.78	4.38	4.45	4.02	8.63
**30xLOQ**	**DOM-1**	**AFG2**	**AFM1**	**AFG1**	**AFB2**	**AFB1**	**HT-2**	**OTB**	**T-2**	**ZEA**
	182.66	18.67	18.60	6.25	7.62	4.70	196.13	34.58	34.57	*
Ccal (ng/mL)	188.11	18.92	19.17	6.50	7.90	4.88	200.25	34.71	33.46	135.05
	194.49	19.27	20.66	6.77	8.30	5.39	207.06	36.52	35.90	116.69
Mean	188.42	18.95	19.48	6.50	7.94	4.99	201.15	35.27	34.64	125.87
RE (%)	4.68	21.03	18.85	27.73	11.77	16.82	11.75	17.56	15.47	4.89
RSD (%)	3.14	1.58	5.45	4.01	4.34	7.13	2.74	3.08	3.53	10.32

**6xLOQ**	**OTA-d$_5$**	**STER**	**NIV**	**DON**	**FUS-X**	**NEO**	**3-ADON**	**15-ADON**	**DAS**
	12.16	6.11	129.02	59.83	40.83	3.50	6.97	11.41	3.73
Ccal (ng/mL)	12.72	6.32	126.20	67.89	50.12	3.77	7.12	11.81	4.14
	12.21	5.90	138.13	61.85	50.45	3.97	8.67	11.50	4.14
Mean	12.36	6.11	131.12	63.19	47.14	3.75	7.59	11.57	4.00
RE (%)	3.02	1.85	7.12	20.77	9.91	21.97	27.76	29.08	4.69
RSD (%)	2.49	3.45	4.76	6.64	11.59	6.36	12.41	1.81	5.83
**30xLOQ**	**OTA-d$_5$**	**STER**	**NIV**	**DON**	**FUS-X**	**NEO**	**3-ADON**	**15-ADON**	**DAS**
	65.10	33.78	643.91	307.70	238.32	19.31	36.29	59.33	20.32
Ccal (ng/mL)	69.22	36.26	577.98	280.64	228.10	18.50	38.94	64.05	21.11
	72.50	39.31	604.88	299.93	245.25	19.30	41.59	66.22	21.12
Mean	68.94	36.45	608.92	296.09	237.22	19.04	38.94	63.20	20.85
RE (%)	14.90	21.50	0.50	13.18	9.32	20.68	25.82	22.55	0.72
RSD (%)	5.38	7.60	5.44	4.71	3.64	2.43	6.81	5.57	2.22

LOQ: limit of quantification; Ccal: calculated concentration; RE: relative error; RSD: relative standard deviation. * not included.
